# A Longitudinal Research on the Distribution and Prognosis of Intracerebral Hemorrhage During the COVID-19 Pandemic

**DOI:** 10.3389/fneur.2022.873061

**Published:** 2022-04-18

**Authors:** Gangqiang Lin, Xueqian Xu, Xiaoqian Luan, Huihua Qiu, Shengfang Shao, Qingsong Wu, Wei Xu, Guiqian Huang, Jincai He, Liang Feng

**Affiliations:** ^1^Department of Neurology, The First Affiliated Hospital of Wenzhou Medical University, Wenzhou, China; ^2^Department of Emergency, The First Affiliated Hospital of Wenzhou Medical University, Wenzhou, China; ^3^Medical Record Room, The First Affiliated Hospital of Wenzhou Medical University, Wenzhou, China; ^4^Outpatient Office, The First Affiliated Hospital of Wenzhou Medical University, Wenzhou, China; ^5^Teaching and Research Section of Epidemiology, The First Affiliated Hospital of Wenzhou Medical University, Wenzhou, China

**Keywords:** intracerebral hemorrhage, COVID-19, longitudinal research, prognosis, lockdown period

## Abstract

**Purpose:**

Globally, intracerebral hemorrhage (ICH) is a common cerebrovascular disease. At the beginning of 2020, due to the coronavirus disease 2019 (COVID-19) pandemic, the allocation of medical resources and the patient treatment and referrals were affected to varying degrees. We aimed to determine the characteristics and prognoses and associated factors of patients with ICH.

**Patients and Methods:**

The baseline demographic characteristics and ICH outcomes were compared between patients diagnosed with ICH between January and June 2020 (the 2020 group) and between January and June 2019 (the 2019 group). COVID-19 positive patients were excluded from the study. A 30-day data from patients in the 2019 and 2020 groups were analyzed to create survival curves for these patients. We also used regression models to identify the significant determinants of poor outcomes [modified Rankin score (mRS): 3–6] and death.

**Results:**

The number of patients diagnosed with ICH was slightly lower in the 2020 group (*n* = 707) than in the 2019 group (*n* = 719). During the lockdown period (February 2020), the admission rates for ICH decreased greatly by 35.1%. The distribution of the patients' domicile (*P* = 0.002) and the mRS (*P* < 0.001) differed significantly between the years. The survival curve revealed that the highest risk of death was in the acute stage (especially in the first 5 days) of ICH. At 30 days, mortality was 19.8% in February 2019 and 29.4% in February 2020 (*P* = 0.119). Multivariate analysis revealed age, baseline mRS, postoperative complications, massive brainstem hemorrhage, and creatinine as factors significantly associated with poor outcomes and death following ICH. Neurosurgery and massive supratentorial hemorrhage were only correlated with the risk of death.

**Conclusion:**

During the lockdown period, the COVID-19 pandemic caused a decrease in the admission rates and severe conditions at admission due to strict traffic constraints for infection control. This led to high mortality and disability in patients with ICH. It is necessary to ensure an effective green channel and allocate adequate medical resources for patients to receive timely treatment and neurosurgery.

## Introduction

Stroke leads to long-term neurological disability and is the second most common cause of death worldwide. Studies have shown that although its incidence, prevalence, and mortality have declined, its overall burden on society has increased (1, 2). Intracerebral hemorrhage (ICH), a subtype of stroke, accounts for 15–20% of all strokes and contributes significantly to the high morbidity and mortality rates worldwide (3, 4). Therefore, the diagnosis and treatment of ICH are necessary.

Coronavirus disease 2019 (COVID-19) and its associated neurological comorbidities (cerebrovascular diseases and cognitive impairment) have posed several challenges for global healthcare (5). The disease was first reported in Wuhan (Hubei Province, China) in December 2019 (6). At the end of January 2020, more than 90,000 people returned to Wenzhou city (located in the south of China; permanent population: 9.25 million) from Wuhan during the Spring Festival. Thus, Wenzhou became one of the most heavily affected areas during the COVID-19 pandemic outside the Hubei Province (7). The prevention and control strategies for the COVID-19 pandemic have led to significant declines in the efficiencies of diagnoses and treatments in hospitals in Wenzhou. Therefore, in this study, we observed the diagnosis and treatment of patients with acute ICH in Wenzhou during the COVID-19 pandemic. We aimed to determine the characteristics and prognoses of these patients in the hope that our data will provide evidence for improved management of ICH during the pandemic.

## Materials and Methods

### Participants and Data Collection

Data for the analysis were obtained from the First Affiliated Hospital of the Wenzhou Medical University, the largest regional medical center for cerebrovascular disease in Wenzhou. It is equipped with a separate neuro-emergency clinic and a mature “green channel” for ICH (this allows the extraction of electronic medical records). We collected the data of participants who were diagnosed with non-traumatic ICH in the neuro-emergency clinic between January and June of 2019 (the 2019 group) and 2020 (the 2020 group). Cases with simple subarachnoid hemorrhage were excluded. First, we recorded the following baseline data from the green channel for ICH: demographic characteristics (age, sex, location of the patient's domicile, and medical histories), data on the stroke event [modified Rankin score (mRS)], clinical history, and physical examination data recorded at first arrival at the emergency room (ER)], amount of time spent in the ER, imaging data (location of the ICH, hemorrhage volume (estimated using the ABC/2 formula)] (4, 8), cerebral computed tomography angiography findings and magnetic resonance imaging findings), and outcomes after observation in the ER. Thereafter, for patients who were transferred to the inpatient departments, additional data were collected through the inpatient records of the neurology department. All patients diagnosed with a stroke in 2020 who were included in our study were followed up 1 month and 1 year later, and the “intercept value” was set as “died”. This retrospective study was approved by the Ethics Committee of the First Affiliated Hospital of Wenzhou Medical University and was performed in accordance with the principles of the Declaration of Helsinki.

Due to the strict epidemic prevention measures in China, patients positive with COVID-19 were excluded from ER and would be sent to the isolation ward to avoid cross-infection. Before admission, a brief inquiry on the epidemiology history of the COVID-19, a blood routine test, and chest CT were demanded to rule out COVID-19 pneumonia from the end of January 2020. From March 2020 till now, nucleic acids and antibodies of COVID-19 could be routinely detected in ER (results could be reported in 2 h for those suspected cases). Therefore, any case that was infected with COVID-19 would be detected quickly and isolated.

The month of February 2020 was defined as the lockdown period. The daily rate of COVID-19 cases was recorded by The Chinese Center for Disease Control and Prevention from 20 January 2020 onwards; partial lockdown measures were implemented on 4 February 2020.

### Definition of Hypertensive ICH

In this study, the diagnosis of hypertension-associated ICH was based on the following: history of hypertension, antihypertensive drug usage in the ER, a systolic blood pressure ≥140 mmHg and/or a diastolic blood pressure ≥90 mmHg (recorded in two or more nursing records from the ER or inpatient department), diagnosis of hypertension at discharge, and exclusion of other definite reasons (such as the moyamoya disease and arteriovenous malformations). The criteria for the diagnosis of a massive ICH were a supratentorial ICH ≥30 ml, cerebellar hemorrhage ≥10 ml, brainstem hemorrhage ≥5 ml or respiratory failure, and intraventricular hemorrhage (or cerebral hemorrhage breaking into the ventricle) requiring emergency surgery. Typical hypertensive hemorrhage is considered to occur at the common site of bleeding, such as basal ganglia, thalamus, brainstem, and cerebellum.

### Prognosis

The clinical outcome measures included the following outcomes [as evaluated by the mRS, in line with criteria of the European Cooperative Acute Stroke Study II): good (mRS: 0–2) and poor (mRS: ≥3, including death) (9). Data on the outcome within the first month from the ICH (30 days) were acquired in different ways. For patients who left the hospital directly after observation in the ER, the data were collected from the nursing records in the ER. For patients who were hospitalized for less than 30 days, the data were obtained from the discharge records or from the routine nursing follow-ups performed within 1 month of the discharge. For patients who were hospitalized for more than 30 days, the data were collected through electronic inpatient records; the 1-year follow-up was conducted through telephonic interviews, where patients were asked questions from a questionnaire. Those unreachable in any way were considered as “lost to follow-up”. The “intercept value” was set as “died”. In the present study, the time of the first ICH episode between January and June 2020 was considered as the starting point of the prognosis.

### Statistical Analysis

Continuous variables were assessed for normality using the Kolmogorov–Smirnov test. Stochastic variables are presented as mean ± SD. Categorical variables were assessed by the Pearson χ^2^ test. A Cox proportion hazards model was used to describe the survival state. The log rank test was used to compare the survival curves. The Cox regression model was used to perform multivariate analysis for the predictors of a poor outcome or death of patients diagnosed in 2020. All statistical analyses were performed using SPSS (version 25.0; IBM, Armonk, NY, USA).

## Results

### Baseline Characteristics of the Patients in the 2019 and 2020 Groups

The 2019 and 2020 groups comprised 719 and 707 patients, respectively. All cases of the 2020 group were tested negative for COVID-19. Through comparative analysis, we found no significant differences in the age (*P* = 0.95), sex (*P* = 0.665), and treatment timeliness (*P* = 0.238) between the two groups. However, the domicile locations (*P* = 0.002) and the composition of mRS (*P* < 0.001) were significantly different between the two groups. There was no significant difference in time spent in the ER between the two groups (*P* = 0.495). The most common cause of ICH was hypertension, and no significant intergroup differences in the incidence of hypertension-associated ICH were observed (2019 vs. 2020 groups: 92 vs. 91%, *P* = 0.567). There was no intergroup significance in the incidence of typical hypertensive hemorrhage (2019 vs. 2020 groups: 80.5 vs. 80.3%, *P* = 0.928) and atypical lobar hemorrhage (2019 vs. 2020 groups: 17% vs. 17.1%, *P* = 0.941). The incidence of massive cerebral hemorrhage was high (2019 vs. 2020 groups: 40% vs. 36%, *P* = 0.273), and most cases were of supratentorial ICH (including of ICH breaking into the ventricles); Moreover, the cumulative mortality rate in the first 30 days was relatively high (2019 group: 22%; 2020 group: 23%), and the total rates of poor prognosis were similar between the two groups (*P* = 0.625; Table 1).

**Table 1:** Clinical characteristics of the patients with ICH diagnosed between January and June 2019 and January and June 2020.

**Parameters**	**The 2019 group** **(*n* = 719)**	**The 2020 group** **(*n* = 707)**	***P-*value**
Age, years	61.32 ± 13.42	61.27 ± 13.55	0.951
Sex, male (%)	494 (69)	494 (70)	0.665
Arrived within 6 h of onset (%)	492 (69)	508 (72)	0.238
Location of domicile (%)			0.002^*^
Main urban area of Wenzhou	193 (27)	197 (28)	
Other city in the Wenzhou area	412 (57)	422 (60)	
Other area in the Zhejiang province	30 (4)	7 (1)	
Other province	84 (12)	81 (12)	
Baseline mRS			<0.001^*^
0–2	122 (17)	36 (5.1)	
3–6	597 (83)	671 (94.9)	
Time spent in the ER (h) (%)			0.392
0 < T ≤ 12 h	277 (39)	259 (37)	
12 h < T ≤ 24 h	198 (28)	180 (26)	
24 h < T ≤ 48 h	145 (20)	169 (24)	
48 h < T ≤ 72 h	59 (8)	62 (9)	
T > 72 h	40 (6)	37 (5)	
Hypertension-associated ICH (%)	661 (92)	644 (91)	0.567
Typical hypertensive hemorrhage (%)	579(80.5)	568(80.3)	0.928
Lobar hemorrhage (%)	122 (17)	121(17.1)	0.941
Massive cerebral hemorrhage (%)			0.273
Supratentorial ICH	241 (33)	223 (32)	
Brainstem hemorrhage	30 (4.2)	30 (4.2)	
Cerebellar hemorrhage	27 (3.8)	19 (2.7)	
Intraventricular hemorrhage	6 (0.83)	13 (1.8)	
Supratentorial and infratentorial hemorrhage	7 (0.97)	3 (0.42)	
Emergency surgery for ICH (%)	121 (17)	123 (17)	0.759
Cumulative mortality rate in the first 30 days, %	157 (22)	162 (23)	0.625

*Data are expressed as mean ± standard deviation or as n (%)*.

*ER, emergency room; ICH, intracerebral hemorrhage; mRS, modified Rankin score*.

* *p values considered statistically significant*.

### Impact of COVID-19 Pandemic on Admission

**Figure 1:**
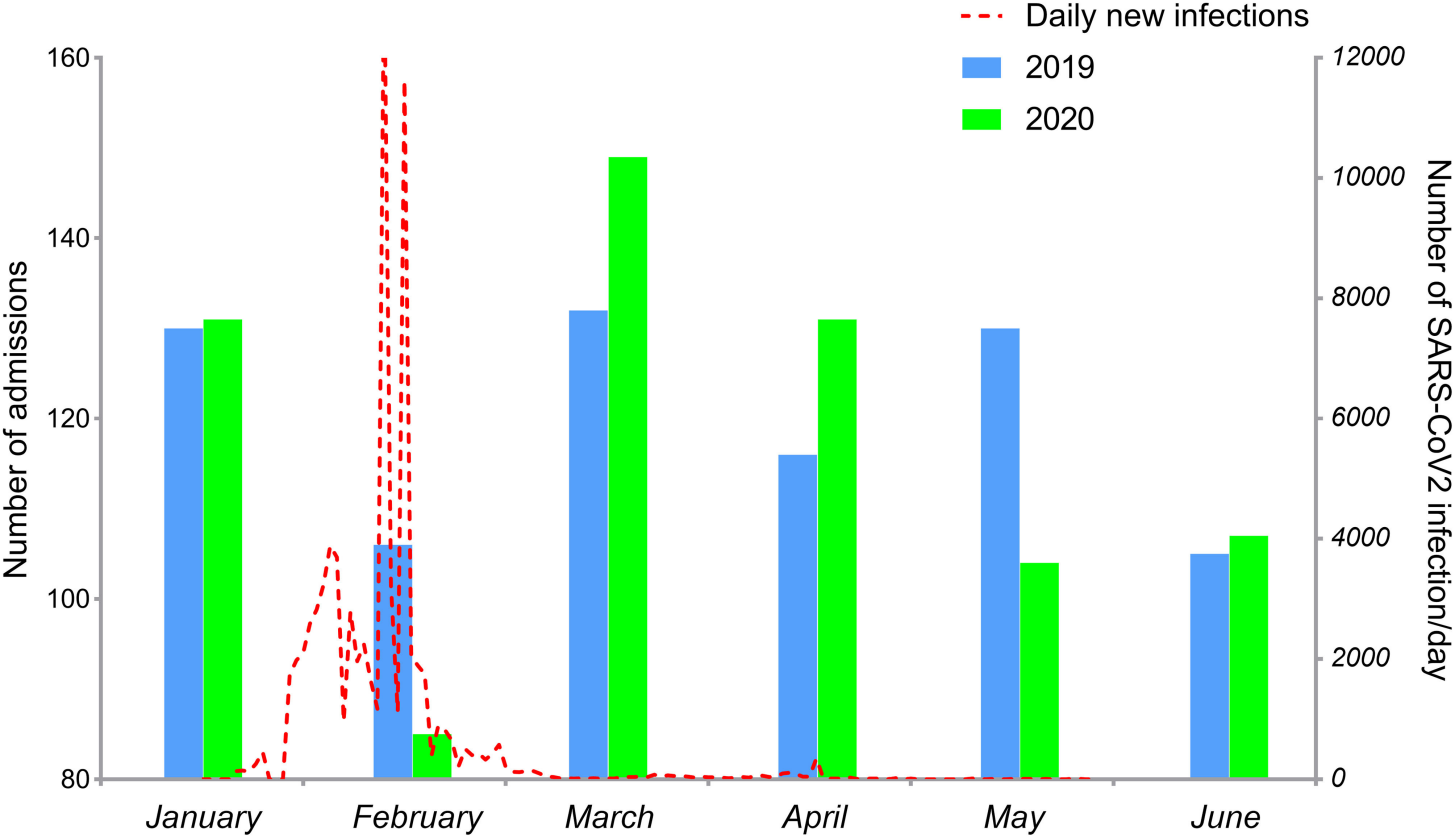
Monthly admission rates for ICH between January and June 2019 (blue column) and January and June 2020 (green column). Daily new COVID-19 cases in China between January 20 and June 30, 2020, (red dotted line).

During the lockdown period (February 2020), the admission rates for ICH decreased significantly by 35.1%, as depicted in Figure 1. After the lockdown period, the total number of cases rebounded in March and April 2020. Due to the COVID-19 outbreak, hospitalizations were strictly controlled in late April 2020 (10), and the total number of patients decreased in May 2020. In June 2020, the number of patients admitted for ICH was similar to the number in 2019.

### Impact of COVID-19 Pandemic on the Mortality and Functional Outcomes

**Figure 2:**
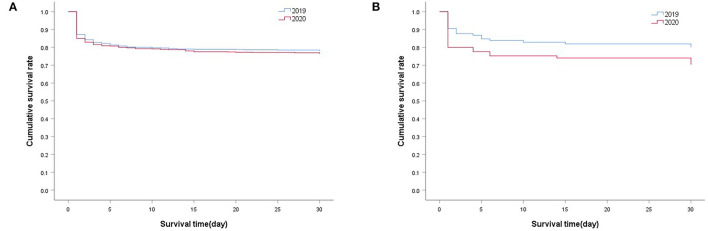
Cox regression analysis of the cumulative survival. **(A)** 30-day survival in 2019 group (*N* = 719) vs. 2020 group (*N* = 707) from January to June, *P* = 0.452; **(B)** 30-day survival of patients in February 2019 (*N* = 106) vs. February 2020 (*N* = 85), *P* = 0.119. The log rank test was used to compare the survival curves.

In the 2020 group, 624 patients were followed up for 1 year; the follow-up rate was 88%. Patients lost to follow-up were younger than those who were followed up (57.70 ± 13.93 years vs. 61.75 ± 13.44, *p* = 0.01); however, no significant differences were noted in the sex ratio (male/female; 60/23 vs. 434/190, *p* = 0.61), baseline mRS (3.93 ± 0.79 vs. 4.05 ± 0.90, *p* = 0.21), and proportion of emergency neurosurgery (16/67 vs. 107/517, *p* = 0.63) between these patient sets. The prognosis was generally unfavorable for ICH. The 1-year mortality for the 2020 group was 27.72% (see Supplementary Figure 1). The highest risk of death for the 2020 group was in the first month after stroke, especially in the first 5 days (Figure 2A). The survival curve showed that 30-day mortality in 2019 and 2020 groups from January to June was almost identical (21.84% vs. 23.48%, *p* = 0.452; Figure 2A). At 30 days, cumulative survival for February 2019 was 80.19%, compared with 70.59% in February 2020 (*p* = 0.119; Figure 2B).

**Figure 3:**
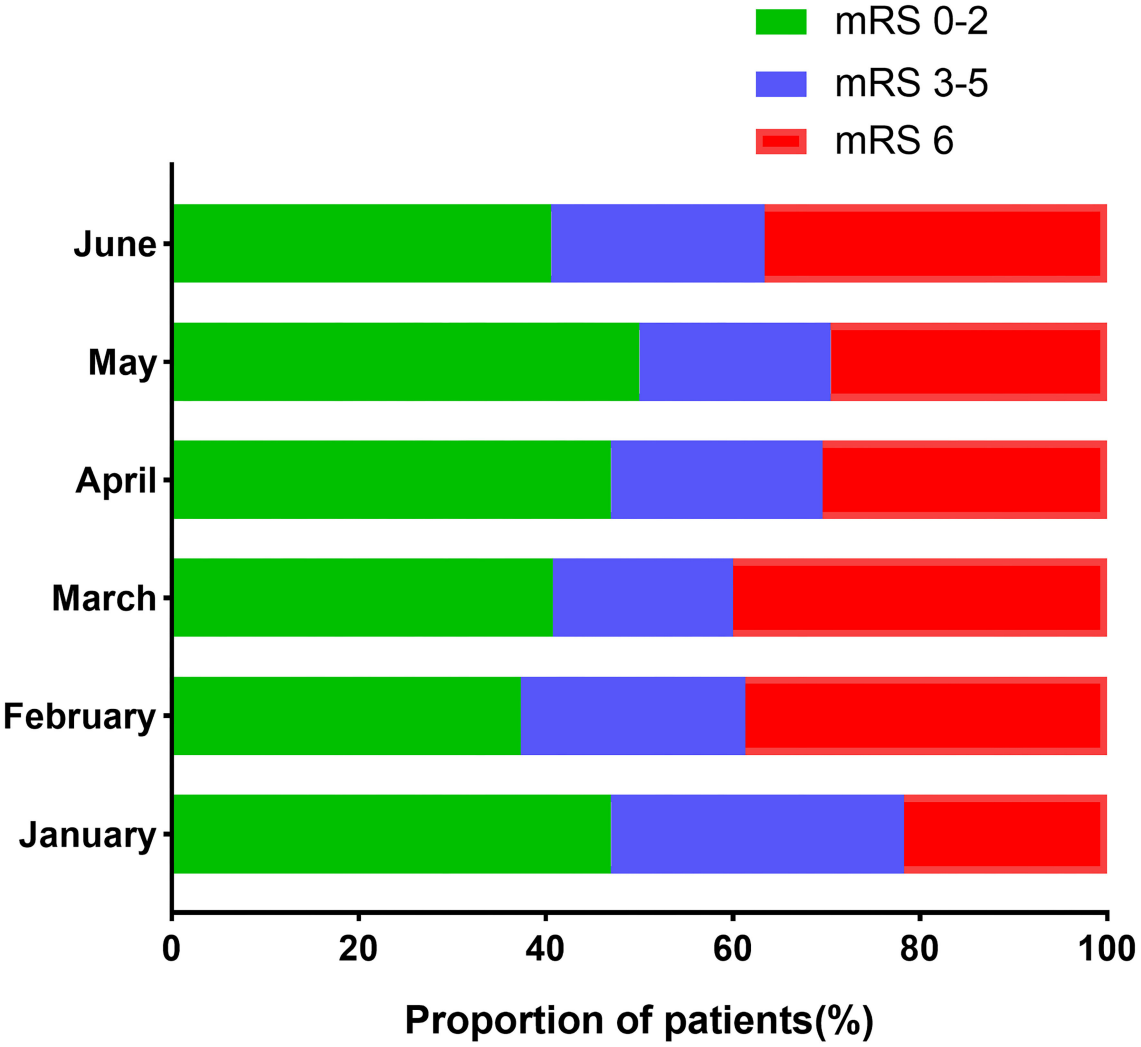
The month-dependent proportion of patients with unadjusted 1-year outcomes in the 2020 group.

Poor outcome events occurred more frequently during the lockdown than during the other months. Patients diagnosed in February 2020 had a less favorable prognosis at 1 year (disability rate: 62.7%, mortality: 38.7%). However, the prognosis did not differ significantly among those diagnosed in the other months. The distribution of the unadjusted 1-year outcome is presented in Figure 3.

### A Significant Determinant of ICH Outcome

The Cox regression model was developed to further determine the predictive value of underlying factors for the outcome of ICH. Multivariate analysis revealed age (HR: 1.021, 95% CI: 1.012–1.030; *p* < 0.001), baseline mRS (*HR*: 2.478, 95% *CI* 2.092–2.935; *p* < 0.001), postoperative complications (*HR*: 2.624, 95% *CI*: 1.495–4.607; *p* = 0.001), massive brainstem hemorrhage (*HR*: 2.825, 95% *CI*: 1.912–4.173; *p* < 0.001), and creatinine (*HR*: 1.001, 95% *CI*: 1.000–1.002; *p* < 0.001) as the factors significantly associated with a poor outcome. Furthermore, multivariate analysis also revealed these factors, namely age (*HR*: 1.017, 95% *CI*: 1,006–1.029; *p* = 0.002), baseline mRS (*HR*: 3.464, 95% *CI*: 2.634–4.554; *p* < 0.001), postoperative complications (*HR*: 6.114, 95% *CI*: 2.886–12.954; *p* < 0.001), massive brainstem hemorrhage (*HR*: 2.392, 95% *CI*: 1.587–3.605; *p* < 0.001), and creatinine (*HR*: 1.001, 95% *CI*: 1.000–1.002; *p* = 0.038), as the factors significantly associated with the risk of death (Table 2). However, neurosurgery (*HR*: 0.174, 95% *CI*: 0.072–0.417; *p* < 0.001) and massive supratentorial hemorrhage (*HR*: 2.287, 95% *CI*: 1.127–4.640; *p* = 0.022) were only associated with the risk of death.

**Table 2:** Multivariate analysis of the primary outcomes of patients in the 2020 group.

**Predictors for**	**Poor outcome**		**Death**	
	**HR (95% *CI*)**	***P-*value**	**HR (95% *CI*)**	***P-*value**
Male	NS	NS	NS	NS
Age (year)	1.021 (1.012–1.030)	<0.001	1.017 (1,006–1.029)	0.002
Baseline mRS (score)	2.478 (2.092–2.935)	<0.001	3.464 (2.634–4.554)	<0.001
Pulmonary infections in the ER	NS	NS	NS	NS
Neurosurgery	NS	NS	0.174 (0.072–0.417)	<0.001
Postoperative complications	2.624 (1.495–4.607)	0.001	6.114 (2.886–12.954)	<0.001
Recurrent stroke	NS	NS	NS	NS
Massive supratentorial hemorrhage	NS	NS	2.287 (1.127–4.640)	0.022
Massive brainstem hemorrhage	2.825 (1.912–4.173)	<0.001	2.392 (1.587–3.605)	<0.001
Massive cerebellar hemorrhage	NS	NS	NS	NS
Creatinine (μmol/L)	1.001 (1.000–1.002)	0.014	1.001 (1.000–1.002)	0.038

*ER, emergency room; ICH, intracerebral hemorrhage; mRS, modified Rankin score; NS, no significance*.

## Discussion

Early detection and intervention are essential for the survival of patients with ICH (11). The COVID-19 pandemic has increased the burdens and challenges faced by healthcare systems worldwide. In the present study, we have provided real-world data of patients diagnosed with ICH during January and June 2020 (i.e., during the COVID-19 pandemic) in Wenzhou (China), and have compared these data with those from the same period in 2019. We observed a significant impact of the pandemic and associated lockdown measures on the admission rates for ICH; a 35.1% decrease was noted in these rates in February 2020. The referenced period is featured with the peak of COVID-19 cases in China; the rate exceeded 3,000 infections/day for the first time on 2 February 2020 and rose to a peak of nearly 14,000 infections/day on 11 February 2020. The rate stabilized to <200 infections/day from 1 March 2020 onwards. A similar study in Germany showed that the admission rates for ICH fell by 42.1% due to an underutilization of the healthcare system (12). Another study conducted in Italy also noted that cases of ischemic stroke seemed to have almost disappeared from the Casualty Department of the authors' institution (13). This situation was observed for not only neurological diseases but also for other diseases, such as ST-segment elevation myocardial infarction (14). Our data, collected from the biggest governmental hospital in Wenzhou, showed that the number of patients diagnosed with ICH reduced significantly after the COVID-19 outbreak; however, this number rebounded in March 2020. Restrictions on traffic would have been an important factor for patients living in villages and towns around Wenzhou, who could have arrived at the hospital originally. An important reason might be a reluctance to seek medical care for the fear of infection, especially in crowded places. Thus, patients would have preferred to go to their local hospital to receive conservative treatment.

We compared whether there were any other differences between the two groups of different years and determined whether these differences were related to the COVID-19 pandemic. There were no significant differences in the sex, age, and timeliness of treatment between the 2019 and 2020 groups. However, we found that the domicile locations of the patients differed significantly between the groups, with only seven patients who were from the non-Wenzhou area of the Zhejiang Province.

The baseline mRS also differed significantly between the two groups; the incidence of mRS ≥3 was higher in the 2020 group than in the 2019 group. We noted that the rates of emergency surgery for ICH and poor outcomes in the ER were also higher in the 2020 group than in the 2019 group (Table 1); however, these differences were not significant. During the pandemic, it was reasonable that more patients with mild ICH chose to be treated locally rather than being sent to our center; the prolonged time for receiving prompt treatment would have caused more severe nerve function damage (15). The most common cause of ICH in both groups was hypertension; therefore, strict management of hypertension is extremely important and imminent for people in the Wenzhou area. Massive cerebral hemorrhage constituted a high proportion of cases in the two groups (43% in the 2019 group and 41% in the 2020 group); supratentorial ICH was the predominant type. There were no significant intergroup differences in the economic state and risk of infection (16, 17). Furthermore, despite traffic restrictions, most patients (72%) arrived at the ER within 6 h of ICH onset in 2020.

The COVID-19 pandemic also affected the patients' outcomes. Our data revealed that the risk of death for patients diagnosed between January and June 2020 was the highest within the first year after ICH, particularly during the first 5 days. According to previous literature, the cumulative mortality rates for ICH were 10.8–31%, 43–54%, and 22.5–71% at 1 month, 1 year, and 5 years after the ICH episode, respectively (18–23). Our data showed a moderate cumulative mortality rate in the first year after ICH (28.9%). Figure 3 shows a comparison of the 1-year mRS, which reflects the long-term functional prognosis for acute ischemic stroke (AIS), between the different months of 2020. It was reasonable to assume that the prognosis improved gradually after the lockdown period. The rebound in June might be attributed to the fluctuation in the COVID-19 cases following a return to work in China.

We also tried to identify the significant determinants of poor post-ICH outcomes and death after adjusting for confounding factors. Age, baseline mRS, postoperative complications, massive supratentorial hemorrhage, and creatinine were significantly associated with a poor prognosis. Hematoma volume seemed to be the supreme risk factor for not only an early outcome but also for long-term prognosis (21, 23, 24). Notably, neurosurgery was a protective factor, whereas massive supratentorial hemorrhage was a harmful factor for the survival of those with large-sized hematomas; however, these factors could not predict further functional outcomes. This observation is in line with those of a previous study conducted in India (25).

The strengths of our study included the large sample size from the Wenzhou medical health system caring for patients, which was the second impaired epicenter during the China epidemic. We used existing infrastructure and expanded with follow-up data collection according to a well-established methodology. Our data demonstrated a relatively high follow-up rate (88%). However, there were still several limitations in our current study. First, data on 1-year survival and functional outcome were only available in the 2020 group. Second, the 1-year outcome was estimated from information provided by the patients or their caregivers and there may be some subjective selection bias. Third, other related factors such as a history of medication with antiplatelet agents or anticoagulants were not acquired. Fourth, the exclusion of COVID-19 positive patients may also contribute to selection bias. Finally, even though there was a trend suggesting that 30-day mortality was higher during the lockdown period, the trend did not achieve significance. Further validation in larger cohorts with longer follow up is warranted.

Overall, the COVID-19 pandemic caused a sudden decline in the admission rates and high mortality and disability rates for ICH in Wenzhou. However, the total number of patients with ICH was almost the same between 2019 and 2020; this suggests a transfer of admissions, rather than missed admissions, in the lockdown period. Therefore, it is important to allocate resources and divert patients when necessary (26, 27). In order to improve the long-term prognosis and survival rates of patients with ICH during the pandemic, ensuring a “green channel” for stroke and allocating adequate medical resources will help in timely neurosurgery. As vaccination becomes more widespread, gradual deregulation of traffic restrictions would effectively improve the prognosis of patients with stroke. Simultaneously, for the high proportion of hypertensive cerebral hemorrhage cases, strengthening the prevention, and control of hypertension before and after ICH is also important (28, 29).

## Data Availability Statement

The original contributions presented in the study are included in the article/Supplementary Material, further inquiries can be directed to the corresponding authors.

## Ethics Statement

The studies involving human participants were reviewed and approved by the First Affiliated Hospital of Wenzhou Medical University. Written informed consent for participation was not required for this study in accordance with the national legislation and the institutional requirements.

## Author Contributions

LF, JH, and GH conceived and designed the study. LF analyzed and interpreted the data. GL and XX collected the basic data, checked the medical records, and investigated the survival state of the patients in the green channel. XL and HQ screened the subjects in the ER. SS checked the nursing records in the ER. QW searched for cases in the online emergency registration system. WX provided advice on the prevention and control of the COVID-19 pandemic. All authors participated in the critical review and editing of the manuscript and approved the final manuscript.

## Funding

The infrastructure for the study was funded by the Wenzhou Municipal Sci-Tech Bureau Programme (Y2020427).

## Conflict of Interest

The authors declare that the research was conducted in the absence of any commercial or financial relationships that could be construed as a potential conflict of interest.

## Publisher's Note

All claims expressed in this article are solely those of the authors and do not necessarily represent those of their affiliated organizations, or those of the publisher, the editors and the reviewers. Any product that may be evaluated in this article, or claim that may be made by its manufacturer, is not guaranteed or endorsed by the publisher.

## Supplementary Material

Supplementary Figure 1 Cox regression analysis of 1-year cumulative survival in the 2020 group (*N* = 624).
