# Supplementary figures and images for: A Longitudinal Research on the Distribution and Prognosis of Intracerebral Hemorrhage During the COVID-19 Pandemic
Source: Front Neurol. 2022 Apr 18;13:873061. doi: 10.3389/fneur.2022.873061 (PMC9062182; doi:10.3389/fneur.2022.873061)

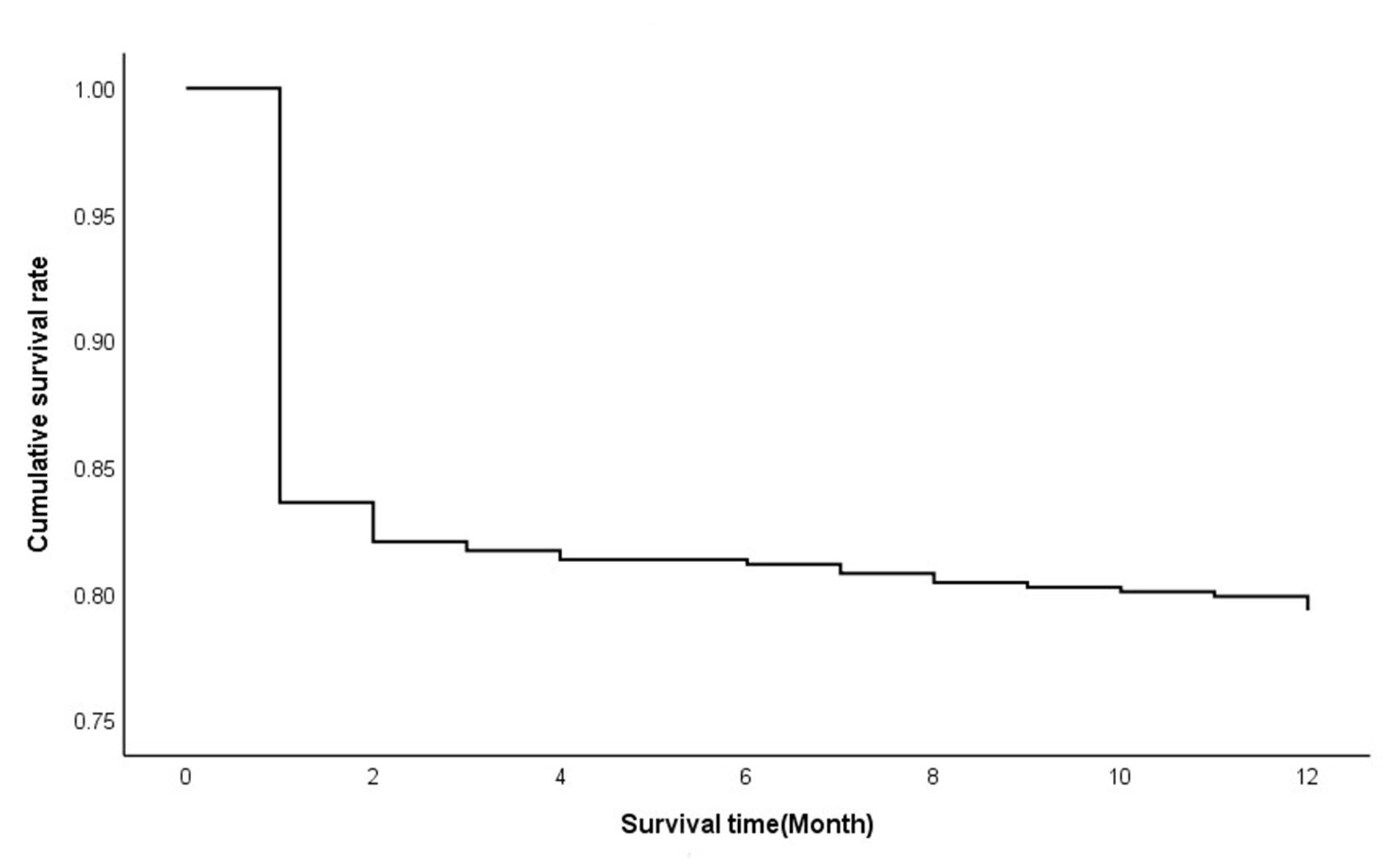

Supplement: Supplementary Figure 1 — Cox regression analysis of 1-year cumulative survival in the 2020 group (N = 624). [file Image_1.JPEG]
